# Social Media–Based Professional Intervention vs Resource Provision for Youth With Suicidal Ideation or Behavior: Protocol for a Randomized Controlled Trial

**DOI:** 10.2196/83303

**Published:** 2026-04-24

**Authors:** Margot Morgiève, Leloup Manon, Anne-Christine Lesniak, Laurine Rougegrez, Nathalie Rigbourg, Vincent Jardon, Marielle Wathelet, Anne-Laure Demarty, Karim Dahache, Alain Duhamel, Thomas Delbarre, Pierre Grandgenèvre, Charles-Edouard Notredame

**Affiliations:** 1 University of Paris CNRS, INSERM CERMES3 Paris France; 2 Groupement d’Étude et de Prévention du Suicide Saint-Benoît France; 3 Psyhub CHU Lille Lille France; 4 Regional Health Agency of Hauts-de-France Lille France; 5 Fédération Régionale de Recherche en Psychiatrie et Santé Mentale des Hauts-de-France Saint-André France; 6 INTERACTION team INSERM U1172 Lille Neuroscience & Cognition Centre Lille France

**Keywords:** social media, adolescent, young adult, suicide prevention, digital, web

## Abstract

**Background:**

Despite high prevalence of suicidal ideation and behavior, adolescents and young adults often underuse mental health services due to both structural and personal barriers. Simultaneously, massive social media (SM) expansion has introduced new mental health threats, while also creating vital spaces where adolescents and young adults can express psychological distress, seek support, and access mental health information. Designed as a bridge to care and a counterbalance to digital risks, the Équipe en Ligne de Prévention du Suicide (ELIOS) project connects adolescents and young adults with suicidal ideation or behaviors with a team of professionally trained web clinicians (nurses or psychologists supervised by a psychiatrist) through SM platforms. ELIOS is currently being evaluated within the Online Referral and Intervention to Prevent Adolescent and Young Adult Suicide (ORIAS) trial to assess its efficacy.

**Objective:**

We assessed the ELIOS system’s efficacy over simple resource provision for adolescents and young adults with suicidal ideation or behaviors.

**Methods:**

ORIAS is a single-center, open-label, 2-arm randomized controlled trial with balanced parallel groups. It will recruit 386 participants aged 18-25 years who spontaneously reach out via the ELIOS Facebook Messenger account with recent suicidal ideation. Eligible adolescents and young adults are randomized to receive either the ELIOS intervention or conventional mental health resources (control group). The ELIOS intervention consists of web-clinicians offering initial support, risk evaluation, counseling, and progressive referral to mental health care. The primary endpoint is the reduction in suicidal ideation intensity at 3 months, measured using the Columbia Suicide Severity Rating Scale. Secondary endpoints include suicide attempts, use of health care services, psychological distress, attitudes toward help-seeking, participant satisfaction, and usage metrics of the intervention at 3 months. The primary endpoint will be compared between the 2 study arms using a constrained longitudinal data analysis model. Secondary endpoints assessed at 3 months will be compared between groups using logistic regression models for binary variables and analysis of covariance for continuous variables.

**Results:**

The ORIAS study was funded in November 2018, and data collection began in October 2022. As of December 2025, 375 participants had been recruited. Recruitment is expected to conclude in April 2026. Data analysis will be conducted between April and December 2026, with the first publication anticipated in early 2027.

**Conclusions:**

ELIOS is the first suicide prevention system embedded directly within the SM environment and daily digital practices of adolescents and young adults. By offering easy, personalized, and timely access to professional help, ELIOS can address help-seeking barriers and reduce suicidality. While the ORIAS trial highlights the inherent challenges of conducting traditional interventional research within dynamic SM settings, it is poised to generate high-quality evidence on the feasibility, acceptability, and effectiveness of digital suicide prevention interventions. These findings could inform and support the broader integration of similar models within public health systems.

**Trial Registration:**

ClinicalTrials.gov NCT04642157; https://clinicaltrials.gov/study/NCT04642157

**International Registered Report Identifier (IRRID):**

DERR1-10.2196/83303

## Introduction

### Context

Globally, suicide is the third leading cause of death among youth aged 15 to 25 years [1]. Depending on the region of the world, between 14.3% and 22.6% of adolescents report experiencing suicidal ideation in the past year, while 4.6% to 16.9% report having attempted suicide at least once [2]. Alarmingly, the situation has significantly worsened over the past 5 years, with a marked rise in suicide attempt rates, particularly among young women, in several European countries [3,4].

Access to appropriate care is widely acknowledged as a key component of suicide prevention, particularly among young populations [5]. However, paradoxically, mental health service use remains low among adolescents and young adults [6,7]. This so-called “service gap,” the discrepancy between a high level of need and limited use of available care [8], has been attributed to both structural and personal barriers [9]. Structural barriers include limited accessibility, high costs, lack of visibility, and inconvenient service delivery [10]. Personal barriers among adolescents and young adults include stigma and self-stigma [11], difficulties in identifying and expressing emotions [12], a self-reliance bias (ie, the tendency to overestimate one's ability to cope independently) [13], and misbeliefs about care providers [14], all of which can significantly hinder help-seeking behaviors [15]. Strikingly, help-seeking has been shown to decrease as psychological distress increases, a phenomenon known as the “help-negation bias” [16].

A major contemporary factor influencing access to care is the extensive digitalization of social life. From now on, every adolescent and young adult younger than 25 years is from the generations Z or alpha, which means they were born and grew up with social media (SM) [17]. In the United States, nearly half of teenagers report being online almost constantly, while in France, 81% connect to a SM platform daily [18]. These platforms have created an unprecedented space for adolescents and young adults to share their struggles and to seek or receive informal support. Searching for mental health information online has become a common practice among adolescents and young adults, and distressed youth often turn to SM to access peer support, alleviate feelings of isolation, and seek emotional assistance [19,20]. Moreover, a significant number of young SM users disclose suicidal thoughts through public online posts [19,21].

Several characteristics of SM communication suggest that it could serve as a powerful lever to help adolescents and young adults overcome classical barriers to seeking mental health support: (1) accessibility helps circumvent geographic constraints and limited availability of local services [22,23]; (2) affordability addresses structural barriers related to cost, facilitating access for financially dependent individuals; (3) timeliness allows users to access support outside of traditional hours aligning with the fluctuating nature of both suicidal ideation severity and the motivation to seek help [24,25]; (4) anonymity and privacy may reduce the confrontational aspect of help-seeking interactions, encouraging emotional expression and self-disclosure [24,26]; (5) the perceived control over interactions provides a sense of safety for individuals who may not be ready for strong interpersonal commitments, as it allows them to disengage from conversations at any time [22].

If the literature acknowledges the original prevention opportunities that the Web has brought out, it also increasingly points out the new or compounded threats to which unregulated interactions or contents can expose [27]. This is the second edge of the sword, as qualified by Robert et al [22]: for instance, SM paved the way for cyberbullying [28], problematic use, at-risk behaviors, and increased dissemination of content with high risk of suicide contagion. Consequently, vulnerable web users exploring the internet with ambivalent intentions would more likely be exposed to normalizing, inciting, or stigmatizing content than to helpful resources.

Given the growing consensus on this issue, researchers and suicide prevention organizations have called for a more proactive use of SM. The aim is to shift the balance so that a suicidal web-user is more likely to encounter support, be guided through help-seeking, and access care than to be pushed toward suicidal behaviors.

Yet, SM platforms remain underused in prevention efforts. Most contemporary information and communication technology–based interventions prioritize forums, websites, mobile apps, or SMS text messaging, whereas the use of SM remains largely confined to awareness-raising initiatives and basic psychoeducational content [29]. For example, Robinson et al [30] co-developed a series of suicide prevention media messages with secondary school students, designed for dissemination on platforms such as Instagram (Meta) and Snapchat (Snapchat Inc). These messages primarily provided contact information for relevant helplines and support services [30]. While such campaigns provide information about available support resources, they do little to address the personal barriers that often prevent adolescents and young adults from seeking care [10,11,31]. Simply put, a young person unable to ask for help offline is unlikely to make a call or book an appointment just because a number appears online.

To address the lack of proactive web-based prevention strategies, we developed the ELIOS system (“Équipe en Ligne de Prévention du Suicide”—online team for suicide prevention). ELIOS is a team of web-clinicians accessible to adolescents and young adults with suicidal ideation or behaviors via SM. Web clinicians operate through a specially designed platform to which several SM channels can converge via their application programming interfaces.

The ELIOS platform was developed by a multidisciplinary team comprising clinical researchers, software developers, and designers coordinated by a project manager. Through an interactive co-design process, the platform was developed and progressively refined based on the needs and feedback expressed by a group of clinicians (nurses and psychologists) and a group of adolescents and young adults with a history of suicidal ideation. Clinicians and adolescents and young adults specifically provided insights into the platform’s user interface and user experience. Importantly, the platform was inspired by the chat systems developed by several helplines in France, most of which were consulted by the project manager. However, because these live chat systems operate in closed digital environments, the project team had to adapt the solution to enable connectivity with SM platforms.

**Figure 1:**
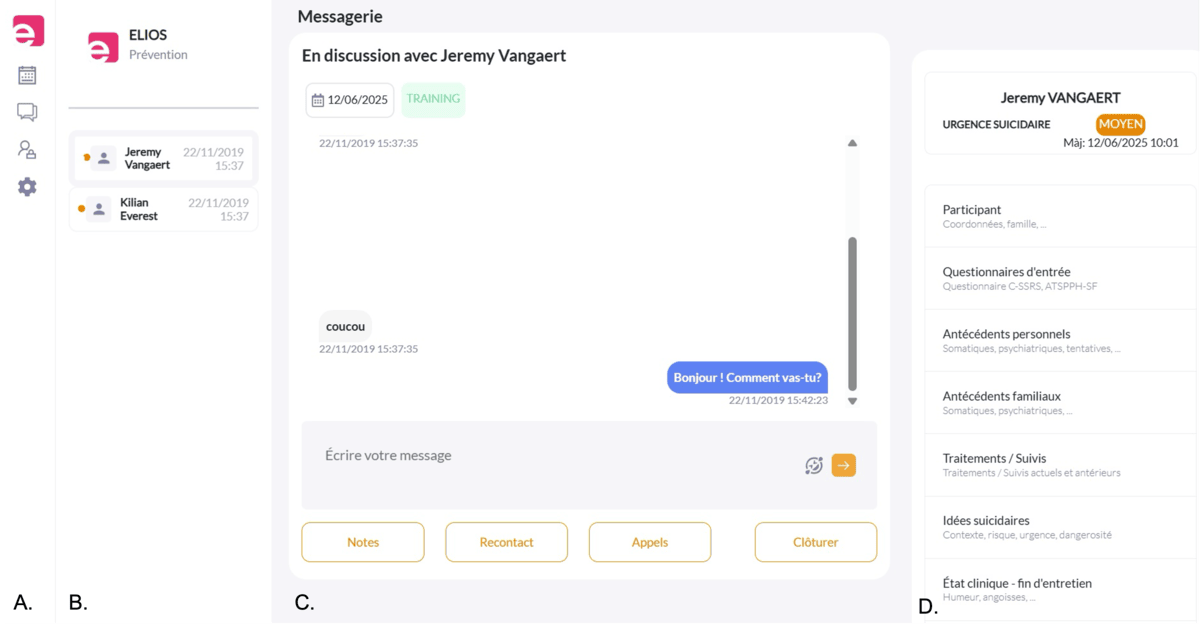
Screenshot of the ELIOS digital platform. (A) Menu allowing the web-clinicians to access the agenda, chat space, list of included participants, and platform setting. (B) Waiting list, prioritized based on waiting time and level of suicidality. (C) Chat space where the web clinicians can leave a note, schedule a follow-up, initiate a call, or close the conversation. (D) Digital clinical record where the web clinician can enter or review information about the participant’s situation, inclusion form results, personal and family medical history, current medications, suicidal ideation, and clinical status.

As shown in Figure 1, the final ELIOS platform consists of a secured webspace with three main components: (1) a chat space where web-clinicians can interact with participants and decide which SM channel to use for communication. Within this space, they can easily access conversation history, switch between channels, or change the participant they are engaging with; (2) a schedule of participants to contact, organized by a hierarchical prioritization algorithm based on simple rules (eg, prioritizing users with high suicidality scores); (3) a digital clinical record that automatically compiles relevant data collected in line with the research protocol (eg, inclusion scale results), while also allowing web-clinicians to manually and ergonomically add any additional information gathered during their interactions with users. Unfortunately, at the time of writing, the platform could only be connected to Facebook Messenger (Meta) due to technical and financial constraints (see Discussion).

Grounded in the understanding that help-seeking is largely driven by intrapersonal factors (37), ELIOS functions as a gateway provider—offering initial support, reinforcing motivation to seek help, and facilitating referral to appropriate services. Recognizing that, for many adolescents and young adults, initiating contact online may feel easier than by phone or in person, ELIOS aims to make the internet a stepping stone toward conventional mental health care.

### Objectives

In this study, we introduce Online Referral and Intervention to Prevent Adolescent and Young Adult Suicide (ORIAS), a randomized controlled trial (RCT) designed to evaluate the efficacy of the ELIOS system.

The primary objective of ORIAS is to demonstrate the superiority of ELIOS in reducing suicidality at 3 months, compared to the simple provision of professional resources, among adolescents and young adults seeking help online for suicidal ideation.

A first set of secondary objectives aims to evaluate the superiority of the ELIOS system over standard professional contact in improving more distal endpoints at three months: (1) reducing the rate of access to conventional health care services, including emergency room visits and hospital admissions following a recent suicide attempt or life-threatening behavior; (2) lowering the incidence of suicide attempts; and (3) decreasing all-cause mortality.

A second set of secondary objectives focuses on exploring potential mediating mechanisms underlying the ELIOS system's effects. This includes assessing its superiority in (4) reducing psychological pain at 3 months; (5) increasing access to mental health care, emergency services, or hospital admissions unrelated to suicide attempts or life-threatening behaviors; and (6) decreasing negative attitudes toward mental health care at 3 months.

Finally, a third set of secondary objectives explores the acceptability and use of the ELIOS system. This involves (7) comparing participant satisfaction at 3 months between the ELIOS intervention and simple professional contact, and (8) measuring platform usage throughout the study period.

## Methods

### ELIOS Intervention

The ELIOS system consists of “web clinicians” who provide direct intervention to adolescents and young adults with suicidal ideation or behaviors from the ELIOS platform via SM channels. These web-clinicians are nurses or psychologists placed under the supervision of a psychiatrist. They are specially trained in clinical care for adolescents and young adults with suicidal ideation or behaviors, including crisis intervention, counseling, motivational guidance, SM engagement, and online textual and iconographic communication.

The goal of the ELIOS intervention is 2-fold: to reduce users’ distress and suicidality, and to ensure an effective referral to mental health services. To achieve this, web-clinicians: (1) provide first-line mental health care, including clinical evaluation and counseling tailored to the participant's estimated suicide risk; and (2) gradually motivate the participant to engage more deeply in care by addressing barriers to help-seeking. As soon as the quality of the therapeutic alliance permits, interactions are transitioned to phone-based communication to enhance the likelihood of successful referral to in-person health care services. Outside of program hours, participants are encouraged to contact 3114, the French national professional suicide prevention helpline, if support is needed [32].

### Study Design

**Figure 2:**
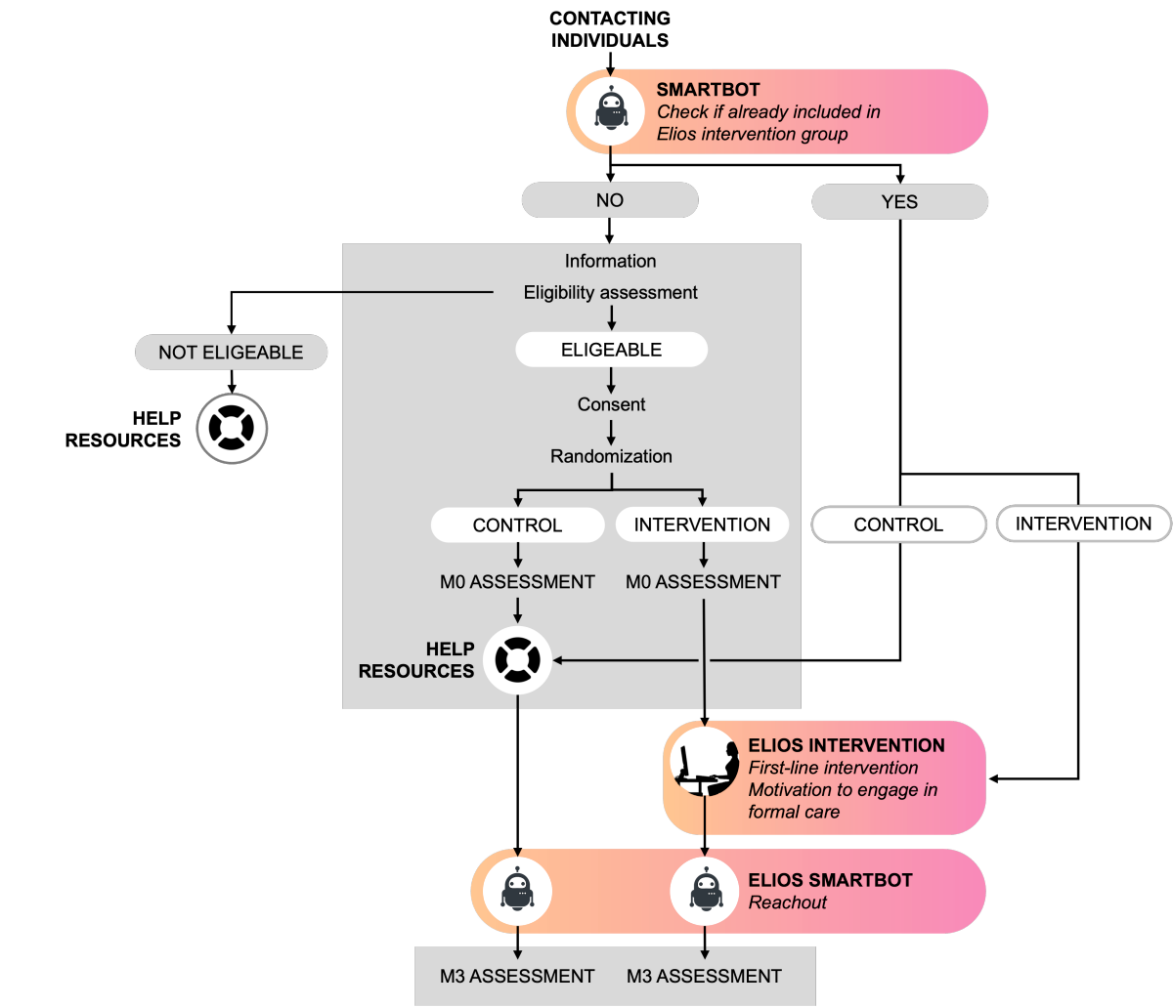
Overview of participant pathway through the Online Referral and Intervention to Prevent Adolescent and Young Adult Suicide protocol.

ORIAS is a randomized, controlled, single-center, open-label, 2-arm, superiority trial with balanced parallel groups. The participant pathway through the study is illustrated in Figure 2. This trial was designed and reported in accordance with the SPIRIT (Standard Protocol Items: Recommendations for Interventional Trials) guidelines. For the complete checklist, refer to Multimedia Appendix 1.

The ELIOS system is promoted through organic and paid SM campaigns, enabling distressed adolescents and young adults to initiate contact spontaneously via its Facebook Messenger account. Upon receiving a first-contact private message, an algorithm-driven smartbot automatically responds through the same channel to begin the interaction. The smartbot verifies whether this is the user’s initial contact, acknowledges their effort in seeking help, and invites them to click a hyperlink to access the ELIOS website. Importantly, the smartbot’s preregistered messages were developed based on input from the focus group of adolescents and young adults.

Once on the website, users are guided through a sequence of pages where they (1) receive comprehensive information about the program and research study, (2) complete an online eligibility assessment form, and (3) if eligible, are invited to provide electronic informed consent. Upon consenting, users proceed to complete online questionnaires for the study’s baseline assessment (M0). Finally, participants are randomly assigned to 1 of 2 study groups. The randomization procedure involves block randomization stratified by sex, with randomly generated blocks of size 4. A random selection among the possible block compositions is then used to generate the final allocation list.

Participants assigned to the control group are directed to a final webpage containing a list of first-line, specialized, and emergency mental health resources, including general practitioners, psychiatric outpatient clinics, emergency services, and the national suicide prevention helpline. The resources are tailored to the participant’s level of suicidality, as assessed through the inclusion forms. This comparator was selected to minimize intervention effects while still providing access to the most reliable mental health resources available online.

Participants assigned to the ELIOS intervention group are also redirected to a final separate page informing them that a web-clinician will contact them as soon as possible via Facebook Messenger to initiate the intervention. If the request is made outside of working hours, the clinician makes initial contact at the beginning of the next working period. Meanwhile, participants are invited to contact 3114, the national suicide prevention helpline, in case of acute distress.

Participants who do not meet the inclusion criteria are also provided with reliable and appropriate resources tailored to their specific situation.

Three months after inclusion, the ELIOS smartbot automatically reaches out to all participants via Facebook Messenger and sends them a hyperlink to the follow-up assessment (M3) through an online form. If the participant does not complete the questionnaire within 1 week, 2 automatic reminders are sent at 1-week intervals. To promote retention, the M3 survey link and reminders are delivered via the same private Facebook Messenger thread used for enrollment, and participation is supported by study compensation (digital gift voucher). Reminder messages are phrased to foster engagement by emphasizing that taking part can contribute to research and ultimately support peers, other young people in distress.

### Participants

To be included, users spontaneously contacting the ELIOS Facebook Messenger account have (1) to be aged 18 to 25 years inclusive, (2) to have been experiencing suicidal ideations in the week prior to the contact, (3) to live in France and speak French, and (4) to provide informed consent. The age limit of 25 years was selected to align with French psychiatric and community services that cater specifically to adolescents and young adults.

### Endpoints

The intensity of suicidal ideation is measured at baseline (M0) and at 3 months (M3). It is assessed using the Intensity of Ideation (IoI) subscale of the Columbia Suicide Severity Rating Scale (C-SSRS) [33]. This subscale comprises 5 items. Participants evaluate different dimensions of their most severe suicidal thoughts during a given period: frequency, duration, controllability, deterrents,and reason for ideation. Each item is rated on a 0 to 5 (controllability, deterrents, and reasons for ideation) or 1 to 5 Likert scale (frequency and duration).

Scores are summed to yield a total ranging from 2 to 25, with higher scores indicating greater intensity of suicidal ideation. The IoI subscale has demonstrated good internal consistency (Cronbach α between 0.73 and 0.95) [33]. The French version used in this study has undergone linguistic validation [34].

All secondary endpoints in both groups are assessed at month 3 (M3) using self-administered questionnaires. Participants are asked: (1) whether and when they consulted a general practitioner, psychiatrist, psychologist, or nurse, or whether they were admitted to a hospital or emergency department for psychological, psychiatric, or substance use-related reasons; (2) whether the consultation or admission was preceded by a suicide attempt or life-threatening behavior; (3) whether and when they attempted suicide without seeking care from conventional mental health services or visiting an emergency department.

Vital status will be determined after the end of the study by querying the Registers of Civil Status, using participants’ identity and place of birth.

Attitudes toward mental health care are assessed using a French version of the Attitudes Toward Seeking Professional Psychological Help–Short Form (ATSPPH-SF) [35]. The ATSPPH-SF is one of the most widely used tools for evaluating attitudes related to help-seeking. It consists of 10 items rated on a 4-point Likert scale from disagree to agree. For 5 items, strong agreement indicates more positive attitudes, while for the remaining 5 items, strong agreement reflects more negative attitudes. We reversed the scoring scale for these latter 5 items so that, overall, a higher ATSPPH-SF score corresponds to a more positive attitude toward seeking help.

Satisfaction of the participants is measured with the Client Satisfaction Questionnaire-8 (CSQ-8). The CSQ-8 is validated and frequently used in relation to mental health services [36]. It consists of 8 items to which the participants respond via Likert scales ranging from 1 (low satisfaction) to 4 (high satisfaction). Higher scores indicate greater satisfaction with the services.

Finally, we collect usage metadata from the platform, including the total number of logins, the number of spontaneous contacts initiated by participants, the mean duration of each interaction, the average number of participants contacted per day, and the rate of successful recontacts (ie, recontacts that received a response).

### Statistical Analysis

The primary endpoint (change in the C-SSRS IoI-subscale score between baseline and 3 months) will be compared between the 2 arms using the constrained longitudinal data analysis model (cLDA) proposed by Liang and Zeger [37]. In the cLDA, both the baseline and postbaseline values are modeled as dependent variables using a linear mixed model, and the true baseline means are constrained to be the same for the 2 treatment arms. Hence, the cLDA provides an adjustment for the baseline values in estimating the treatment effects. The treatment effect will be estimated by the time-by-arm interaction, including gender as a covariate.

The secondary binary endpoints at 3 months will be compared between the 2 arms using logistic regression models adjusted for gender. Odds ratios and their 95% CI values will be derived from logistic regression models as effect sizes (experimental vs control). The secondary quantitative endpoints will be compared between the 2 arms using analysis of covariance to adjust for baseline score and gender. Standardized mean differences and their 95% CI values will be calculated as the effect size. If the model residuals are not distributed normally (despite the log-transformation of the data), analysis of covariance will be performed on the rank-transformed data (nonparametric analysis).

Finally, the variables related to the use of the platform will be described in the intervention group. These quantitative variables will be expressed as mean (SDs), median (IQR), and ranges.

Statistical tests will be performed with a 2-tailed alpha risk of 0.05. No adjustment for multiple testing will be applied, and thus, all secondary objectives will be considered exploratory. All analyses will be performed in all randomized patients based on their original group of randomization, according to the intention-to-treat, after handling missing values.

### Required Sample Size

According to the results from Nam et al [38], we assume that the 3-month mean score at the C-SSRS lol-subscale will be 9.8 (SD 5.4) in the control arm. In the ELIOS intervention arm, we expect this value to be decreased by 20%, corresponding to a mean difference of 2 points at 3 months. With an SD of 5.4 points, a type I error of 5%, and a power of 90%, we calculated that 154 patients per arm will be required to detect a difference of 2 points in the primary endpoint with a 2-sided *t* test. To account for an anticipated loss to follow-up rate of 20%, we will enroll 193 participants per group, which means a total of 386 participants.

### Ethical Considerations

Participation in the ORIAS study was contingent upon the signing of informed consent by all participants. As compensation for their participation, they received a €20 (US $23) digital gift voucher. Required measures have been implemented under the supervision of the data protection officer of the CHU de Lille to ensure compliance with the General Data Protection Regulation guidelines. Only data that are strictly necessary for the research purposes, as predefined in the authorized protocol, are collected. All data are gathered via the secure ELIOS platform or website. Access to individual-level information stored on the platform is restricted to authorized web-clinicians and protected by personal, 2-factor–authenticated, password-secured accounts. Data can be extracted by the research team only in anonymized form, ensuring that all analyses are conducted on fully anonymous datasets. All data are hosted on a dedicated, secure virtual private health data server at the CHU de Lille, France. Based on these measures, the ORIAS study was approved by the Sud-Ouest and Outre-Mer IV Committee for the Protection of Persons (IORG0009855) and authorized by the French Data Protection Authority (Commission Nationale de l’Informatique et des Libertés). The trial was registered on ClinicalTrials.gov under the identifier NCT04642157.

## Results

The ORIAS study was funded in November 2018, and data collection began on October 12, 2022. As of December 2025, a total of 375 participants had been enrolled, 190 in the intervention group and 185 in the control group, representing 97% of the required sample size. Overall, 50% (187/375) of participants were male (87/190, 46% in the intervention group and 100/185, 54% in the control group). The mean age was 21.6 (SD 2.3) years in the overall sample (intervention: 21.7, SD 2.4 years; control: 21.5, SD 2.33 years). Due to irregular enrollment rates, participant recruitment is expected to conclude in April 2026. Data analysis will be conducted between April 2026 and the end of 2026, and the first publication is anticipated in early 2027. No deviations from the registered protocol were observed.

## Discussion

### Originality and Contribution of the ORIAS Protocol

To our knowledge, ELIOS is the first suicide prevention helpline fully tailored to the SM environment and the digital behaviors of adolescents and young adults. By harnessing the interactive features of SM platforms, ELIOS aims to overcome the help-seeking barriers that often prevent distressed adolescents and young adults from accessing traditional support. Given that RCTs of SM-based interventions remain scarce and challenging to conduct, the ORIAS study will generate novel, high-quality evidence not only regarding ELIOS but also for the wider field.

### Comparison With Previous or Ongoing Work

The potential of SM as a tool for suicide prevention, particularly among adolescents and young adults, has been envisaged for a decade [39-41], with several systematic reviews suggesting promising effectiveness [19,42,43]. However, progress in this area remains understudied compared to the research on the risks associated with SM. Most efforts have primarily focused on risk detection via data mining, psychoeducational dissemination, peer-to-peer support, online training, or efforts to improve mental health literacy [19,29]. Evidence-based, professionally led applications remain scarce [44], with only a few notable exceptions. These include the REFRAME-IT program [45] which delivers cognitive behavioral therapy to at-risk secondary school students via a secure digital platform; the Rebound project [46], which integrates positive psychology, peer support, and psychosocial interventions for adolescents and young adults recovering from a severe depression; and the Safe Conversations program [30] which facilitates supervised discussions within a closed Facebook group for secondary school students in communities recently affected by youth suicides. All 3 programs complement traditional therapy by offering remote, evidence-based interventions to preidentified at-risk adolescents and young adults who opt to engage in dedicated digital spaces. In contrast, ELIOS adopts a universal prevention model. As recommended by Montague et al [47], it leverages both the technological and socio-interactional affordances of SM platforms to fill gaps in mental health service accessibility.

### Expected Results

Seamlessly integrated into the everyday digital environments of adolescents and young adults, ELIOS aligns with their habitual online behaviors, enabling them to reach out easily and spontaneously. This design facilitates the delivery of timely, context-sensitive support by trained web-clinicians, which we anticipate will reduce the intensity of suicidal ideation among distressed users [48]. Beyond providing immediate relief, ELIOS is also structured to engage adolescents and young adults through minimal initial interaction, creating opportunities to enhance motivation and ultimately increase the likelihood of accessing professional support. Through these proximal outcomes, we expect ELIOS to contribute to a reduction in suicide attempts and deaths among participants.

### Methodological and Ethical Challenges

Despite some initiatives, a clear gap remains in best-practice guidelines and standardized frameworks for both digital interventions and research in this domain [40]. As a result, like many pioneering teams, we encountered significant early-stage challenges in implementing the ORIAS study, often navigating uncharted territory.

One key challenge shared by most researchers in this field [49] involved securing and maintaining the necessary ethical and regulatory approvals. Both the ELIOS intervention and the ORIAS study raise complex ethical considerations. For example, when a web-clinician initiates contact, the participant is anonymous. This anonymity, coupled with the inherent limitations of online interactions, complicates risk assessment and constrains the ability to implement emergency interventions [40]. Nevertheless, ELIOS is built on the premise that it provides a critical preventive touchpoint for individuals who might otherwise never seek help. To mitigate the risks of missed intervention opportunities, ELIOS is explicitly designed not as a stand-alone solution but as a gateway to traditional health care. Accordingly, web-clinicians focus on fostering participant engagement with the aim of gradually reducing anonymity and facilitating direct access to professional support. Secondly, confidentiality concerns are frequently raised in relation to SM–based interventions. Within the ELIOS system, several safeguards have been implemented to address these issues: all conversations are conducted exclusively through Facebook’s private messaging feature, a data protection officer ensures that data collection and storage processes are fully compliant with the General Data Protection Regulation guidelines, and web clinicians operate under strict ethical protocols. Nonetheless, ELIOS remains exposed to a particular vulnerability regarding data confidentiality: under European legislation, data shared on SM platforms is co-owned by both the user and the platform provider. As a result, we have no oversight or control over how Meta may access, process, or retain the data exchanged during interactions. Considering this unavoidable limitation, and in recognition of the need to balance potential confidentiality risks against the urgent imperative to reduce life-threatening risk, the French data protection authority (Commission Nationale de l’Informatique et des Libertés) still granted authorization to proceed with the study. To mitigate potential risks associated with data sharing on the Meta, web clinicians are instructed to transition to phone-based interactions as early as possible in the exchange to secure communications. In line with ELIOS’ objectives, textual interactions are intended to be limited to initial engagement and to guide users as quickly as feasible toward more formal and more data-secure support modalities. Importantly, prior to providing informed consent, participants receive clear and explicit information that textual interactions occur within the private space of the SM channel used and remain under their own responsibility.

Another limitation of the ELIOS system is that it is accessible solely via Facebook Messenger, a platform less frequently used by adolescents and young adults. This arose primarily from technical and economic constraints. The more widely adopted platforms among Generation Z in France [50], TikTok (ByteDance Ltd.), Instagram, and Snapchat, either lack compatible communication interfaces, automatically reject integration attempts, or permit access only through paid services beyond our financial capacity. Nevertheless, our recruitment rate remains promising, likely since nearly 40% of individuals aged 18-25 years still use Facebook [50] and because we promoted ELIOS via Instagram.

Likewise, due to research regulations, we were required to exclude minor participants from the ORIAS study. This exclusion represents a significant missed opportunity for both prevention and research, as younger adolescents are among the most affected by suicidal ideation and attempts, and simultaneously among the heaviest users of SM [51].

### Implications for Research and Prevention Practices

Most of ORIAS’s limitations stem from a mismatch between SM interventions and the rigor and demands of RCTs. Additionally, these limitations arise from the lack of acculturation of academic stakeholders to digital fields, including their codes and norms, which sometimes results in overly stringent positions. Despite these challenges, we remained committed to complying with ORIAS’s requirements to determine whether ELIOS or similar innovations merit inclusion in the current prevention arsenal. Specifically, generating high-level evidence would provide a compelling argument to persuade decision-makers to implement ELIOS as the SM channel for the French national suicide prevention helpline (3114), to encourage SM platforms to grant broad access to ELIOS, and to convince regulatory authorities to authorize ELIOS’s use by minors.

### Conclusions and Future Perspectives

The regulation of SM access, particularly for minors, is a topic of global discussion. If proven effective, ELIOS could offer a complementary preventive approach by providing innovative access to professional support. If combined with data-mining techniques used to identify at-risk users, it could also serve as a practical tool for developing a proactive suicide prevention outreach system. However, this would require a more in-depth examination of specific ethical challenges.

## Appendix

Multimedia Appendix 1 SPIRIT checklist.

## Abbreviations

ATSPPH-SF: Attitudes Toward Seeking Professional Psychological Help–Short Form
cLDA: constrained longitudinal data analysis
CSQ-8: Client Satisfaction Questionnaire-8
C-SSRS: Columbia Suicide Severity Rating Scale
ELIOS: Équipe en Ligne de Prévention du Suicide
IoI: Intensity of Ideation subscale
ORIAS: Online Referral and Intervention to Prevent Adolescent and Young Adult Suicide
RCT: randomized controlled trial
SM: social media
SPRIIT: Standard Protocol Items: Recommendations for Interventional Trials

## Data Availability

Only the promoter and investigators will access the final trial dataset. The datasets used and/or analyzed during this study are available from the corresponding author on reasonable request.
