# Supplementary material for: Social Media–Based Professional Intervention vs Resource Provision for Youth With Suicidal Ideation or Behavior: Protocol for a Randomized Controlled Trial
Source: JMIR Res Protoc. 2026 Apr 24;15:e83303. doi: 10.2196/83303 (PMC13156537; doi:10.2196/83303)
Supplement: Multimedia Appendix 1 [file resprot_v15i1e83303_app1.pdf]

SPIRIT

STANDARD PROTOCOL ITEMS: RECOMMENDATIONS FOR INTERVENTIONAL TRIALS

SPIRIT 2013 Checklist: Recommended items to address in a clinical trial protocol and related documents*

Section/item	Item No	Description	Addressed on page number
**Administrative information**			
Title	1	Descriptive title identifying the study design, population, interventions, and, if applicable, trial acronym	Paper, p1,6
Trial registration	2a	Trial identifier and registry name. If not yet registered, name of intended registry	Paper, p13
	2b	All items from the World Health Organization Trial Registration Data Set	Infra, p7
Protocol version	3	Date and version identifier	Infra, p7
Funding	4	Sources and types of financial, material, and other support	Paper, 23
Roles and responsibilities	5a	Names, affiliations, and roles of protocol contributors	Paper, 1,24
	5b	Name and contact information for the trial sponsor	Paper, 1
	5c	Role of study sponsor and funders, if any, in study design; collection, management, analysis, and interpretation of data; writing of the report; and the decision to submit the report for publication, including whether they will have ultimate authority over any of these activities	Paper, 23
	5d	Composition, roles, and responsibilities of the coordinating centre, steering committee, endpoint adjudication committee, data management team, and other individuals or groups overseeing the trial, if applicable (see Item 21a for data monitoring committee)	Infra, p7-9

**Introduction**			
Background and rationale	6a	Description of research question and justification for undertaking the trial, including summary of relevant studies (published and unpublished) examining benefits and harms for each intervention	Paper, 4-6
	6b	Explanation for choice of comparators	Paper, 9
Objectives	7	Specific objectives or hypotheses	Paper, 7
Trial design	8	Description of trial design including type of trial (eg, parallel group, crossover, factorial, single group), allocation ratio, and framework (eg, superiority, equivalence, noninferiority, exploratory)	Paper, 9
**Methods: Participants, interventions, and outcomes**			
Study setting	9	Description of study settings (eg, community clinic, academic hospital) and list of countries where data will be collected. Reference to where list of study sites can be obtained	Paper, 9-10
Eligibility criteria	10	Inclusion and exclusion criteria for participants. If applicable, eligibility criteria for study centres and individuals who will perform the interventions (eg, surgeons, psychotherapists)	Paper, 10
Interventions	11a	Interventions for each group with sufficient detail to allow replication, including how and when they will be administered	Paper, 7-9
	11b	Criteria for discontinuing or modifying allocated interventions for a given trial participant (eg, drug dose change in response to harms, participant request, or improving/worsening disease)	NA
	11c	Strategies to improve adherence to intervention protocols, and any procedures for monitoring adherence (eg, drug tablet return, laboratory tests)	Paper, 10
	11d	Relevant concomitant care and interventions that are permitted or prohibited during the trial	NA
Outcomes	12	Primary, secondary, and other outcomes, including the specific measurement variable (eg, systolic blood pressure), analysis metric (eg, change from baseline, final value, time to event), method of aggregation (eg, median, proportion), and time point for each outcome. Explanation of the clinical relevance of chosen efficacy and harm outcomes is strongly recommended	Paper, 10-12
Participant timeline	13	Time schedule of enrolment, interventions (including any run-ins and washouts), assessments, and visits for participants. A schematic diagram is highly recommended (see Figure)	Paper, 9-10

Sample size	14	Estimated number of participants needed to achieve study objectives and how it was determined, including clinical and statistical assumptions supporting any sample size calculations	Paper, 10
Recruitment	15	Strategies for achieving adequate participant enrolment to reach target sample size	Paper, 9

Methods: Assignment of interventions (for controlled trials)

Allocation:			
Sequence generation	16a	Method of generating the allocation sequence (eg, computer-generated random numbers), and list of any factors for stratification. To reduce predictability of a random sequence, details of any planned restriction (eg, blocking) should be provided in a separate document that is unavailable to those who enrol participants or assign interventions	Paper, 9
Allocation concealment mechanism	16b	Mechanism of implementing the allocation sequence (eg, central telephone; sequentially numbered, opaque, sealed envelopes), describing any steps to conceal the sequence until interventions are assigned	Paper, 9
Implementation	16c	Who will generate the allocation sequence, who will enrol participants, and who will assign participants to interventions	Paper, 9
Blinding (masking)	17a	Who will be blinded after assignment to interventions (eg, trial participants, care providers, outcome assessors, data analysts), and how	NA
	17b	If blinded, circumstances under which unblinding is permissible, and procedure for revealing a participant's allocated intervention during the trial	NA

Methods: Data collection, management, and analysis

Data collection methods	18a	Plans for assessment and collection of outcome, baseline, and other trial data, including any related processes to promote data quality (eg, duplicate measurements, training of assessors) and a description of study instruments (eg, questionnaires, laboratory tests) along with their reliability and validity, if known. Reference to where data collection forms can be found, if not in the protocol	Paper, 9,10
	18b	Plans to promote participant retention and complete follow-up, including list of any outcome data to be collected for participants who discontinue or deviate from intervention protocols	Paper, 10

Data management	19	Plans for data entry, coding, security, and storage, including any related processes to promote data quality (eg, double data entry; range checks for data values). Reference to where details of data management procedures can be found, if not in the protocol	Paper, 9
Statistical methods	20a	Statistical methods for analysing primary and secondary outcomes. Reference to where other details of the statistical analysis plan can be found, if not in the protocol	Paper, 12,13
	20b	Methods for any additional analyses (eg, subgroup and adjusted analyses)	Paper, 12,13
	20c	Definition of analysis population relating to protocol non-adherence (eg, as randomised analysis), and any statistical methods to handle missing data (eg, multiple imputation)	Paper, 12,13
**Methods: Monitoring**			
Data monitoring	21a	Composition of data monitoring committee (DMC); summary of its role and reporting structure; statement of whether it is independent from the sponsor and competing interests; and reference to where further details about its charter can be found, if not in the protocol. Alternatively, an explanation of why a DMC is not needed	Infra, p10
	21b	Description of any interim analyses and stopping guidelines, including who will have access to these interim results and make the final decision to terminate the trial	NA
Harms	22	Plans for collecting, assessing, reporting, and managing solicited and spontaneously reported adverse events and other unintended effects of trial interventions or trial conduct	Infra, p11-12
Auditing	23	Frequency and procedures for auditing trial conduct, if any, and whether the process will be independent from investigators and the sponsor	Infra, p12-13
**Ethics and dissemination**			
Research ethics approval	24	Plans for seeking research ethics committee/institutional review board (REC/IRB) approval	Paper, 13
Protocol amendments	25	Plans for communicating important protocol modifications (eg, changes to eligibility criteria, outcomes, analyses) to relevant parties (eg, investigators, REC/IRBs, trial participants, trial registries, journals, regulators)	Infra, p13

Consent or assent	26a	Who will obtain informed consent or assent from potential trial participants or authorised surrogates, and how (see Item 32)	Paper, 10
	26b	Additional consent provisions for collection and use of participant data and biological specimens in ancillary studies, if applicable	NA
Confidentiality	27	How personal information about potential and enrolled participants will be collected, shared, and maintained in order to protect confidentiality before, during, and after the trial	Paper, 9,10
Declaration of interests	28	Financial and other competing interests for principal investigators for the overall trial and each study site	NA
Access to data	29	Statement of who will have access to the final trial dataset, and disclosure of contractual agreements that limit such access for investigators	Paper, 23
Ancillary and post-trial care	30	Provisions, if any, for ancillary and post-trial care, and for compensation to those who suffer harm from trial participation	NA
Dissemination policy	31a	Plans for investigators and sponsor to communicate trial results to participants, healthcare professionals, the public, and other relevant groups (eg, via publication, reporting in results databases, or other data sharing arrangements), including any publication restrictions	Infra, p13
	31b	Authorship eligibility guidelines and any intended use of professional writers	Paper, 24-25
	31c	Plans, if any, for granting public access to the full protocol, participant-level dataset, and statistical code	Paper, 23
**Appendices**			
Informed consent materials	32	Model consent form and other related documentation given to participants and authorised surrogates	P16-21
Biological specimens	33	Plans for collection, laboratory evaluation, and storage of biological specimens for genetic or molecular analysis in the current trial and for future use in ancillary studies, if applicable	NA

*It is strongly recommended that this checklist be read in conjunction with the SPIRIT 2013 Explanation & Elaboration for important clarification on the items. Amendments to the protocol should be tracked and dated. The SPIRIT checklist is copyrighted by the SPIRIT Group under the Creative Commons "Attribution-NonCommercial-NoDerivs 3.0 Unported" license.

All items from the World Health Organization Trial Registration Data Set

Primary Registry and Trial Identifying Number: NCT04642157

Date of Registration in Primary Registry: 2020-07-13

Secondary Identifying Numbers:

- 2018_93
- 2020-A00252-37 (Other Identifier) (OTHER: ID-RCB number,ANSM)
- PHRCN-18-0418 (Other Identifier) (OTHER: DGOS number, PHRC-N)

Source(s) of Monetary or Material Support:

The ORIAS protocol was funded by the French Ministry of Health. The development of the ELIOS system was supported by the Sisley-d'Ornano Foundation, the Crédit Agricole Nord de France Foundation, the CCAH, the Clinea Company, the MACSF Foundation, and the Fondation de France.

The sponsors had no role in study design; collection, management, analysis, and interpretation of data; writing of the report; and decision to submit the report for publication.

Primary Sponsor: CHU Lille

Contact for Public Queries: charles-edouard.notredame@chu-lille.fr

Contact for Scientific Queries: charles-edouard.notredame@chu-lille.fr

Public Title : Online Referral and Intervention to Prevent Adolescent and Young Adult Suicide (ORIAS)

Scientific Title: Social media-based professional intervention (ELIOS) vs. resource provision for suicidal youth: a randomized controlled trial

Countries of Recruitment: France

Health Condition(s) or Problem(s) Studied: suicidal ideation

Recruitment Status: recruiting

Expected Completion date: 2026

Intervention(s), Key Inclusion and Exclusion Criteria, Study Type, Date of First Enrollment, Sample Size, Primary Outcome(s), Key Secondary Outcomes, Ethics Review: cf. article

Date and version identifier: September, 6th 2018

Composition, roles, and responsibilities of the coordinating centre, steering committee, endpoint adjudication committee, data management team, and other individuals or groups overseeing the trial, if applicable (see Item 21a for data monitoring committee) :

In a multi-disciplinary perspective, the ORIAS project involves different research team of complementary skills.

In a multi-disciplinary perspective, the ORIAS project involves different research teams with complementary skills. PSYchic team, SCALab, CNRS UMR9193, France

The PSYchic team has proven solid expertise in clinical and epidemiological research, especially in the realm of suicidology. In a research-action perspective, it develops dual skills in implementing broad-scope prevention programs and conducting specific trials to evaluate these programs. The PSYchic team is strongly committed to the national strategy for suicide prevention (86). In particular, it carries out or contributes to the assessment of multi-regional programs such as VigilanS (see Section 3.2.1) and Papageno (see Section 10.6.1).

Based on its experience, the PSYchic team coordinates in close interweaving the implementation and functioning of the ELIOS system, as well as the conduct of the ORIAS trial, which implies

- Supervision of the ELIOS system
  - Supervision of the platform design and implementation (ongoing)
  - Coordination of the recruitment, training, and clinical supervision of the web-clinicians
  - Verification of the conformity of the ELIOS intervention with clinical standards
  - Centralization of significant event reports
- Supervision of the ORIAS trial
  - Monitoring of the study progress and respect of the schedule
  - Monitoring of the inclusion rates
  - Carrying out 3-month phone reminders, by an investigator blind to the group of allocation

- Verification of the conformity of the study with regulatory norms
- Control of the securing and access to the databases
- Promotion of the results (communication and publication)

As the PSYchic team **is** responsible for the trial, it also coordinates the respective contributions of all other teams and programs.

The Centre d'Investigation Clinique (CIC) of Lille, France

The CIC of Lille has strong regulatory, methodological, and logistical experience, based on more than 30 studies led in close partnership with psychiatric teams. It provides crucial contributions to obtaining and successfully implementing 8 Programmes Hospitaliers de Recherche Clinique.

The CIC contributes to the general organization and coordination of the research and handles its logistic aspects. It also helps ensure compliance with regulatory standards and good clinical practices.

The Biostatistics Department of the CHU Lille, France

The Biostatistics Department of the CHU Lille brings together methodologists, biostatisticians, lawyers, and biologists to support the research team of the CHU Lille, boost their projects, increase the scientific soundness and methodological rigor of their work, and optimize the probability of successful implementation and promotion of their studies.

In ORIAS, the Biostatistics Department of the CHU Lille is in charge of cleaning and managing the databases and of carrying out the statistical analysis.

The Fédération Régionale de Recherche en Psychiatrie et Santé Mentale des Hauts-de-France (F2RSM Psy)

The F2RSM Psy (www.f2rsmpsy.fr) is a regional structure that federates the research activities of all psychiatry and mental health facilities in the Nord – Pas-de-Calais region (extension to the Hauts-de-France Region is under progress). It plays an important role in initiating, supporting, and valorizing clinical and epidemiological trials. It actively promotes interdisciplinarity by recruiting and facilitating collaboration among researchers from different fields. It also develops a strong culture of combining territorial anchoring (as close as possible to population needs) and national reach.

The F2RSM Psy hosts several ORIAS contributors, thus facilitating their communication and coordination: Margot Morgiève, sociologist, who has strong expertise in co-design methodology of new technologies with individuals suffering from severe mental health disorders and suicidal behaviors, the Papageno program, and part of the VigilanS program (see Section 10.6.1). The F2RSM Psy also shares its knowledge of territorial resources in France to facilitate the intervention of the ELIOS web-clinicians.

<u>Investigators</u>

Investigator	Grade	Discipline	Facility	City	Country	Contact
Dr Charles-Edouard Notredame	M.D.	Child and adolescent psychiatry	CHU Lille	Lille	France	charles-edouard.notredame@chru-lille.fr<br>+33 3 20 44 45 84
Pr Guillaume Vaiva	M.D.<br>Ph.D.	Adult psychiatry	CHU Lille	Lille	France	guillaume.vaiva@chru-lille.fr<br>+33 3 20 44 45 84
Dr Debien Christophe	M.D.	Adult psychiatry	CHU Lille	Lille	France	christophe.debien@chru-lille.fr<br>+33 3 20 44 48 48
Dr Grandgenèvre Pierre	M.D.<br>Ph.D.	Adult psychiatry	CHU Lille	Lille	France	pierre.grandgenevre@chru-lille.fr<br>+33 3 20 44 44 60
Dr Briffault Xavier	Ph.D.	Sociology	Cermes3	Paris	France	briffault.xavier@wanadoo.fr<br>+33 1 49 58 36 36
Dr Morgiève Margot	Ph.D.	Sociology	F2RSMPsy	Lille	France	margotmorgieve@yahoo.fr<br>+33 6 09 59 65 32
Mr Stéphane Duhem	M.S.	Psychology	CIC,<br>CHU Lille	Lille	France	stephane.duhem@chru-lille.fr<br>+33 3 20 44 68 91

Study coordination commitee

Dr Charles-Edouard Notredame
Coordinating clinical investigator
Child and Adolescent Psychiatry Department
Hôpital Fontan - CHU Lille
2, Rue Verhaeghe, 59037 Lille Cedex
Email: charles-edouard.notredame@chru-lille.fr

Guillaume Vaiva
Scientific Supervisor Adult Psychiatry Department
Hôpital Fontan - CHU Lille
2, Rue Verhaeghe, 59037 Lille Cedex

Pr Alain Duhamel
Methodologist Health Research Department
CHU Lille
6, rue du Pr Paul Laguesse, 59037 Lille Cedex

Mr. Edouard MILOIS Coordinnating CRA
Head of operational Team
Direction de la Recherche et del'Innovation,
6 rue du Pr Laguesse, 59037 Lille cedex

Mrs. Laetitia DELASSUS Coordinnating CRA
Head of Promotion Technical and Regulatory Team
Direction de la Recherche et de l'Innovation,
6 rue du Pr Laguesse, 59037 Lille cedex

Mr Karim DAHACHE Project Manager
Technical and
Regulatory Team
Direction de la Recherche et de
l'Innovation,
6 rue du Pr Laguesse, 59037 Lille cedex

Composition of data monitoring committee (DMC); summary of its role and reporting structure; statement of whether it is independent from the sponsor and competing interests; and reference to where further details about its charter can be found, if not in the protocol. Alternatively, an explanation of why a DMC is not needed

Research data is collected, managed, and exploited in strict compliance with the present protocol and in respect of the French Regulation on Clinical Research. This implies

- Specific authorization request to the CNIL before any collection
- Online data collection through the ELIOS website
- Storage at the CHU Lille, France, on secure servers physically located in access-controlled rooms, and backed up nightly
- Analysis in accordance with the methodology described in MR 06001 of the CNIL, in the Biostatistics Department of CHU Lille, under the supervision of Pr Alain Duhamel

The research database is locked after the end of the last participant's follow-up.

Clinical data is recorded for the sake of the participants' care, web-clinicians' supervision, and improvement of ELIOS services. Collection, management, and exploitation are submitted to the French Regulation on Health Data, which implies

- Specific authorization request to the CNIL before any collection
- Unsystematic collection of Clinical Identity Card information by the web-clinicians, systematic recording of the phone conversations in the form of auditory files, and systematic recording of textual interactions. Textual records consist of duplicates of the contents of the social media private channels used to interact with the participants
- Information to the participants that textual interactions remain under their responsibility on the private space of the social media channel used to interact
- Protection of access to the platform by private personal passwords
- Hosting on a dedicated secured virtual private health data server at the CHU Lille, France
- Limitation of communication with website users to SSL-protected HTTPS protocol to protect passwords and data in transit over the internet
- Possibility to extract all or part of the anonymized database

Access to data is restricted to individuals who are directly involved in the study. Data may be modified by any physician participating in the study or a fellow working with a physician participating in the study, subject to the agreement of the CCI.

Aria Developing a monitoring committee was not useful in this protocol for the following reasons

- There is no specific therapeutic intervention
- There is a negligible risk of complications related to the study

The contact procedure and possibility to deliver rescue and/or psychotherapeutic intervention represent a clear benefit for the participants.

Plans for collecting, assessing, reporting, and managing solicited and spontaneously reported adverse events and other unintended effects of trial interventions or trial conduct

Description of Safety Assessment Parameters and Protocol Risks

Because the protocol focuses on suicidal behaviors—and although it belongs to the Type 2 research category—it paysclose attention to all possible adverse events that may occur during the 3-month follow-up.

The only potential risk expected is that participants may perceive the intervention and protocol as intrusive, which couldalter their compliance with care.

Expected adverse events include:

- Psychopathologic symptoms or disorders (e.g., major depressive episode, anxiety disorder, suicidal ideation)
- Suicides
- Deaths from any cause
- Suicide attempts
- Psychiatric hospital admissions

At any point, the ORIAS web-clinician provides psychotherapeutic support to participants presenting with distress.

Adverse Events Collection and Reporting

Responsibilities of the Investigator

For each adverse reaction, the investigators assess both the severity and the causal relationship with study-specific acts or protocol procedures. Only serious adverse reactions potentially related to the study or protocol are notified to the sponsor.

Investigators also notify the concerned care team's vigilance unit of any adverse reactions unrelated to the study.
The investigators ensure follow-up of adverse reactions.
They promptly notify the sponsor (Cellule Vigilance du Pôle Promotion Interne de la Direction de la Recherche en Santé) of any new safety data by fax at 03 20 44 57 11.

Responsibilities of the Sponsor

In the event that new safety data emerges during the study, the sponsor immediately sends a declaration of this new data, along with any measures taken, to the CPP and ANSM via e-mail.

Notification of Serious Adverse Events (SAEs)

Theoretically, a Type 2 study does not require systematic SAE collection. However, since some SAEs are of interest, the investigators notify the sponsor without delay upon becoming aware of the following events:

- Suicides
- Deaths from any cause
- Suicide attempts
- Psychiatric hospital admissions

These SAEs must be reported using the SAE Form included in the case report file. This form is sent to the sponsor (Cellule Vigilance du Pôle Promotion Interne de la Direction de la Recherche en Santé) by fax at 03 20 44 57 11.

For each SAE, the investigators:

- Document the event report with as much detail as possible
- Provide follow-up information to the sponsor
- Attach the following documents anonymously, whenever possible:
    - A copy of the hospitalization report
    - A copy of all relevant complementary exam results
    - Any other document they consider useful and relevant

Frequency and procedures for auditing trial conduct, if any, and whether the process will be independent from investigators and the sponsor

To ensure accurate data collection, laboratory notebooks will be checked regularly by the sponsor's Clinical Research Associate (CRA). During trial site monitoring visits CRA will have free access to

- laboratory notebooks with data from patients included in the study
- medical files and nursing files for these patients
- the investigator's notes

This protocol has been assigned to a Type 2 interventional study category. The goal of monitoring will therefore be to

- confirm that patients are enrolled and have signed the informed consent for
- confirm that inclusion criteria are respected
- confirm the main primary end-point
- follow and report SAE
- identify any unexpected events that may require amendment

Plans for communicating important protocol modifications (eg, changes to eligibility criteria, outcomes, analyses) to relevant parties (eg, investigators, REC/IRBs, trial participants, trial registries, journals, regulators)

Substantial modifications" refer to changes that significantly influence any aspect of the trial—particularly the protection of patients participating in the trial, including their safety, the validity of the study, the quality and safety of the tested products, and the interpretation of the scientific documents explaining the study phases or operating procedures.

The sponsor submits any request for a substantial modification to the Ethics Committee for approval.
As soon as approval is received, the sponsor transmits an amended version of the protocol to the investigators.

Plans for investigators and sponsor to communicate trial results to participants, healthcare professionals, the public, and other relevant groups (eg, via publication, reporting in results databases, or other data sharing arrangements), including any publication restrictions

<u>To the general public</u>

The summary results of the ORIAS trial will be available on the ELIOS website, with an effort to make them accessible to any lay reader. If conclusive, the findings will be actively promoted to funders and competent health policy-makers in order to advocate for a long-term implementation of ELIOS as a common-low health system, accessible to the whole population. In case ELIOS actually becomes such a sustained and open component of the national prevention strategy, the results of the ORIAS trial would be widely spread to the population in order to promote access to the system and increase its populational efficacy. This nation-wide communication campaign, conceived as an integral part of the prevention strategy, would be carried out under the expertise of the Papageno program. Of note, it would considerably boost the prevention actions based on the detection or signaling of suicidal posts on social media (already available on Twitter and Facebook, for example), as it will meet the need for professional interactive online resources to provide to distressed users.

<u>To the scientific community</u>

Publications

Publication in scientific journals will follow a pre-defined plan, including, at least

- Publication of the method to provide visibility to the ELIOS prevention system and to the ORIAS protocol, but also to lay solid basis for the subsequent publications
- Publication of the main results, coordinated by the CCI and signed by all the co-investigators
- Publication of ancillary results, coordinated by the CCI, to which all co-investigators may not systematically participate, and to which other authors may contribute

All articles will be submitted to impacted journals, with a decremental strategy (i.e. by submitting the articles to the most relevant journals in a decreasing order with respect to their impact-factor, starting from the relevant journal with the highest impact factor). Most publications – especially those dealing with the main results – will be submitted to international journals, However, we will pay attention to give visibility to the program in France by submitting at least one publication in a French impacted journal. Targeted journals may include generalist, psychiatry, public health, suicidology, or psychology journals.

Communications

Communications related to the ELIOS methodology and/or to the ORIAS findings will be submitted to the main national and international congresses. This may include:

- National congresses of psychiatry: Encéphale, Congrès Français de Psychiatrie (CFP), Congrès Français de Psychiatrie et de Neurologie de Langue Française (CPNLF), Société de l'Information Psychiatrique (SIP)
- International congresses of psychiatry: European Psychiatric Association (EPA) annual congress
- National congresses of suicidology: Geps annual congress
- International congress of suicidology: congress of the International Association for Suicide Prevention (IASP), European Symposium on Suicide & Suicidal Behavior (ESSB)

Communication may be delivered by any of the co-investigator and collaborators, subject to the CCI's approval.

Model consent form and other related documentation given to participants and authorised surrogates

INFORMATION LETTER
For participants in the research involving human subjects entitled:
"Study of the effectiveness of a digital help and online intervention platform for suicide prevention among adolescents and young adults"
ID-RCB: 2020-A00252-37
Sponsor: Lille University Hospital (CHU de Lille)
(ORIAS Study No. 2018_93)
Principal Investigator: Dr. Charles-Edouard Notredame
Fontan Hospital, CHU de Lille, 59037 LILLE Cedex – France – Phone number: +33 (0)3 20 30 23 40

Dear Sir or Madam,

This document describes the study you are being invited to take part in. It summarizes the currently available information and answers common questions regarding your participation in this research.

We invite you to participate in a biomedical research protocol focused on suicide prevention through a web platform called ELIOS.

Indeed, understanding and addressing this issue remains a major public health challenge.

STUDY OBJECTIVES

The goal of this study is to test the ELIOS web platform in the context of suicidal ideation, to assist adolescents and young adults in such situations and promote access to care.

ELIOS is a web platform that adolescents and young adults can easily access through various social media channels to seek help when experiencing suicidal thoughts. This research invites you to participate via this platform.

STUDY PROCEDURE

Before anything else, you are provided with this information letter. You will have a reflection period to decide whether or not to participate in the study.

If you have further questions or would like more information, you can contact the research team at orias@chru-lille.fr, and one of the study investigators will get back to you to address any concerns.

If you agree to participate in the study, you will be asked to provide online consent via electronic signature by checking the box "I consent" at the end of this letter on the consent form.

The electronic signature is equivalent to a handwritten one. It confirms your identity and the integrity of signed documents.

Once consent is signed, you will complete questionnaires on your mental health status, suicidal ideation, and use of tobacco, alcohol, or drugs. You’ll also be asked about any previous access to care.

Then, a random assignment will determine which of the two intervention procedures you’ll receive, both administered through the ELIOS platform:

- Procedure A is the one being tested in this study. It connects you with a team of health professionals (nurses or psychologists) via the social network of your choice.
- Procedure B is the standard procedure. It provides you with contact details and resources to use if needed.

In both cases, you will be contacted again after 3 months via your chosen social network to complete the same questionnaires regarding your mental health, suicidal thoughts, access to care, and substance use over the past 3 months.

Your responses to these questionnaires are essential to the success of this study.

Your total participation in the study will therefore last 3 months.

EXPECTED BENEFITS

We hope the support procedures evaluated in this study may help you personally and encourage access to care if needed. We also hope ELIOS will serve as a gateway to mental health services for people who might otherwise be unable to seek help due to existing barriers.

RISKS AND INCONVENIENCES

Seeking help through the ELIOS platform poses no major risks. The main inconvenience is the time required to answer the questionnaires. If any questions make you uncomfortable, you are free to stop answering at any time.

ALTERNATIVES AND END OF PARTICIPATION

Participation in this study does not prevent you from seeking help as you normally would and does not affect any care that may already be in place. Ending participation early will not impact your ongoing medical care.

ELIGIBILITY CONDITIONS

To participate in this research, you must be between 18 and 25 years old and live in France.

Participation requires informing your general practitioner, unless you object.

ETHICAL AND REGULATORY ASPECTS

Your participation is entirely voluntary. You may refuse or withdraw at any time without giving any reason and without affecting your standard care or the quality of services you receive.

The investigator may also stop your participation at any time for medical, administrative, or other reasons.

You are not required to make an immediate decision and may take the time you need.

Lille University Hospital is authorized to conduct this research. As required by law, this study received favorable approval on XX/XX/XXXX from the Comité de Protection des Personnes (an independent ethics committee tasked with safeguarding participant safety). In addition, Lille University Hospital has taken out liability insurance for this study (SHAM – 18 rue Edouard Rochet – 69372 Lyon cedex 08 – Contract No. 144.893).

The sponsor and investigator will conduct this research in accordance with the protocol, international Good Clinical Practice (GCP) guidelines, and applicable laws and regulations.

CONFIDENTIALITY – PERSONAL DATA

In the context of this research, your personal data will be processed to analyze the results in line with the study's stated objectives.

Medical and other relevant data will be shared with the study sponsor and any collaborators, including entities in or outside the EU, provided the receiving country offers adequate data protection as recognized by French authorities. Your data will be coded (identified only by a study number and initials).

Data may also be securely shared with health authorities in France.

According to the General Data Protection Regulation (GDPR 2016/679) and French data protection laws, you have the following rights:

* Right of Access (Art. 12 GDPR): You may access your health data at any time during or after the study.
* Right to Information (Art. 15 GDPR): You may request details about how your data are collected, processed, and shared.
* Right to Rectification (Art. 16 and 19 GDPR): You may request corrections to inaccurate personal data.

* Right to Erasure (Art. 17 and 19 GDPR): You may request deletion of data no longer needed for the study.
* Right to Restrict Processing (Art. 18 and 19 GDPR): You may, under certain conditions, limit how your data are used.
* Right to Data Portability (Art. 20 GDPR): You may request to receive your personal data in a transferable format or have them sent to another entity.
* Right to Object (Art. 21 GDPR): You may object to the processing of your personal data at any time unless there are overriding legitimate reasons.

Consent and withdrawal of consent:
Your data can only be processed with your consent (Art. 6 GDPR). You may withdraw consent at any time (Art. 7.3 GDPR).

To exercise your rights, contact the study investigator or the sponsor's Data Protection Officer (DPO).

You also have the right to file a complaint with the CNIL (French data protection authority):
https://www.cnil.fr/fr/webform/adresser-une-plainte

Data Protection Officer (DPO) Contact:
Guillaume DERAEDT – CHU de Lille
Data Protection Officer (CIL/DPO)
2 avenue Oscar Lambret, 59037 LILLE CEDEX
Email: guillaume.deraedt@chru-lille.fr

CNIL Contact:
Commission Nationale de l'Informatique et des Libertés
3 Place de Fontenoy TSA 80715, 75334 PARIS CEDEX 07

Research data will be retained for at least 15 years under the responsibility of CHU de Lille.

There are no additional costs to you for participating in this study.

STUDY RESULTS

Global results and publications will be shared with you once the study is completed and the data are available.

FOR ANY QUESTIONS

You may ask any questions and request additional information at any time before, during, or after the study by contacting:

Dr. Charles-Edouard Notredame
Psychiatrist and Principal Investigator
Fontan Hospital, CHU de Lille, 59037 LILLE Cedex – France
Phone: +33 (0)3 20 30 23 40
Email: orias@chru-lille.fr

Thank you for taking the time to read this information letter.
Please check this box to confirm that you have read all 3 pages of the information letter:
